# Report of the Delegates to the Convention of Colleges of Pharmacy

**Published:** 1870-12

**Authors:** 


### REPORT OF THE DELEGATES TO THE CONVENTION OF COLLEGES OF PHARMACY.

The undersigned, according to instructions, representing the Chicago College of Pharmacy, at the Convention of Colleges, held in Baltimore, Sept. 14, beg leave to submit the following report:

The Convention met in the hall of the Baltimore College of Pharmacy. Mr. Joseph Roberts, of Baltimore, was duly elected President, and Prof. J. Farris Moore, also of Baltimere, was chosen Secretary.

The following colleges and associations were represented: The Maryland College of Pharmacy; the Colleges of New York, Massachusetts, and Chicago; the Pharmaceutical Associations of California and New Jersey.

On motion, Drs. Squibb, Hoffman, and Stabler, and Mr. Milhau, who were present, were invited to seats in the Convention, and to participate in the deliberations. The object of the meeting being to confer upon the subject of pharmaceutical education, and a uniform standard of graduation of students. After a prolonged discussion, the following recommendatory resolutions were adopted:

1. That, in the opinion of this meeting, more attention to the preliminary education of those who propose to enter the business of pharmaceutists is needed, and it is earneslty recommended to the Colleges and Societies of Pharmacy to urge their members, and the profession generally to give greater care to this subject in taking apprentices.

2. That a term of four years' service in a dispensing drug store be recommended to be exacted from students in pharmacy before coming up for examination.

3. That apprentices should not be taken under sixteen years of age, and shall be twenty-one years of age before being entitled to receive their diplomas.

4. That the branches to be taught in Colleges of Pharmacy should at least include lectures on general chemistry, elementary botany, materia medica, and the general facts and principles of pharmacy; and, when practicable, opportunity should be provided for general and analytical chemistry.

5. That it is inexpedient, at the present time, to recommend any uniformity in conduction of the examination for graduation, but it is earnestly recommended that whatever method of examination be adopted, should include questions both oral and written, and that particularly a familiarity with the physical properties of specimens should be insisted on.

6. The diplomas should not be recognized as evidence of qualification, unless based on four years' service in a dispensing shop.

7. That each College of Pharmacy be requested to take action on these resolutions and report next year.

This organization of delegates was, on vote, made permanent, and is to meet annually, at the same time and place as the American Pharmaceutical Association.

The meeting then adjourned.

Respectfully submitted.

ALBERT E. EBERT, *Chairman.*
THOMAS WHITFIELD.
E. H. SARGENT.
N. PIERPONT.

